# Prediction of drop-out and functional impairment in recent-onset schizophrenia spectrum disorders

**DOI:** 10.1192/j.eurpsy.2021.123

**Published:** 2021-08-13

**Authors:** A. Mucci, P. Bucci, I. Winter Van Rossum, C. Arango, L. Baandrup, B. Glenthøj, P. Dazzan, A. Demjaha, P. Mcguire, C. Martínez Díaz-Caneja, S. Leucht, R. Rodriguez-Jimenez, R. Kahn, S. Galderisi

**Affiliations:** 1 Department Of Psychiatry, Univeristy of Campania Luigi Vanvitelli, Naples, Italy; 2 Department Of Psychiatry, University Medical Center Utrecht Brain Center, Utrecht University, Utrecht, Netherlands; 3 Child And Adolescent Department Of Psychiatry, Hospital General Universitario Gregorio Marañón, Madrid, Spain; 4 Department Of Clinical Medicine, University of Copenhagen, Copenhagen, Denmark; 5 Department Of Psychosis Studies, Institute of Psychiatry, Psychology and Neuroscience, King’s College, London, United Kingdom; 6 Department Of Psychosis Studies, King’s College London, London, United Kingdom; 7 Psychiatry, Hospital General Universitario Gregorio Marañón, Madrid, Spain; 8 Department Of Psychiatry And Psychotherapy, School of Medicine, Technical University Munich, Munich, Germany; 9 Psychiatry, 12 de Octubre Hospital Research Institute, CIBERSAM, Madrid, Spain; 10 Psychiatry, The Mount Sinai Hospital, New York, United States of America

**Keywords:** First-episode schizophrenia, Functional Outcome, Persistent negative symptoms

## Abstract

Persistent negative symptoms are associated with worse outcome in both first-episode and chronic subjects with schizophrenia. The identification of these symptoms in recent-onset subjects is still controversial as retrospective data are often unavailable. The prospective assessment of persistence of negative symptoms might represent a valid alternative but the length of the persistence is still to be established. The present study investigated the prevalence of negative symptoms of moderate severity, unconfounded by depression and extrapyramidal symptoms at baseline in a large cohort of patients in the early stage of a schizophrenia-spectrum disorder, recruited to the OPTiMiSE trial. Persistent unconfounded negative symptoms were assessed at 4, 10 and 22 weeks of treatment. Symptomatic remission, attrition rate and psychosocial functioning was evaluated in subjects with short-term (4 weeks) persistent negative symptoms (PNS) and in those with negative symptoms that did not persist at follow-up and/or were confounded at baseline (N-PNS). Negative symptoms of moderate severity were observed in 59% of subjects at baseline and were associated to worse global functioning. PNS were observed in 7.9% of the cohort, unconfounded at both baseline and end of 4-week treatment. PNS subjects showed lower remission and higher attrition rates at the end of all treatment phases. Fifty-six percent of subjects completing phase 3 (clozapine treatment) had PNS, and 60% of them were non-remitters at the end of this phase. The presence of short-term PNS during the first phases of psychosis was associated with poor clinical outcome and resistance to antipsychotic treatment, including clozapine.

**Disclosure:**

Prof Mucci has been a consultant and/or advisor to or has received honoraria from Gedeon Richter Bulgaria, Janssen Pharmaceuticals, Lundbeck, Otsuka, Pfizer and Pierre Fabre.

